# Microcosm evaluation of saturation and labile carbon controls on nitrate persistence in vadose zone soils

**DOI:** 10.3389/fmicb.2026.1975842

**Published:** 2026-10-28

**Authors:** Liangyao Chen, Xiang Liu, Zhaohuan Li, Han Li, Yunqiang Xu

**Affiliations:** School of Environment, Tsinghua University, Beijing, China

**Keywords:** groundwater table recovery, microbial functional potential, nitrate dynamics, nitrogen source, vadose zone soil, water filled pore space

## Abstract

Groundwater table recovery increases water saturation in the lower vadose zone, altering nitrate persistence. To clarify how soil properties and substrate availability modulate these dynamics, two microcosm experiments compared static water-filled pore space levels (50%, 80%, and 100%) and various nutrient additions in a Beijing soil profile. Between days 1 and 45, nitrate remained stable at 50% WFPS but declined by 39.15%~53.52% at 100% WFPS, with the largest reduction in the deepest soil due to higher initial organic carbon. Glucose addition enhanced the 45-day decline to 85.30% by inducing reducing conditions (-89 mV) within the first 15 days. In contrast, ammonium inputs persisted as the dominant dissolved form, while glycine triggered a sequential cascade marked by organic nitrogen decrease and transient ammonium, nitrite, and nitrate peaks. Day 45 sequencing confirmed that glucose and glycine shifted bacterial structures and gene profiles. Although gaseous measurements or isotope tracing are required to resolve specific pathways, these findings demonstrate that labile carbon governs the extent of nitrate decline under high saturation, while substrate composition shapes inorganic nitrogen accumulation timing.

## 1 Introduction

Groundwater depletion has become a major challenge to water security and ecosystem stability in many intensively exploited aquifer systems (Perrone and Jasechko, 2019; Famiglietti and Ferguson, 2021). Interbasin water transfer and interventions that reduce abstraction or enhance recharge have created opportunities for groundwater recovery in previously overexploited regions (Zhuang, 2016; Jasechko, 2026). In Beijing, reduced groundwater abstraction together with the South-to-North Water Diversion Project has reversed the long-term decline in groundwater levels (Li et al., 2025). This hydrological transition raises additional questions about groundwater quality, given the widespread occurrence of nitrate contamination in China and its relevance to aquifers in the Beijing region (Jiang et al., 2022; Zhou et al., 2024).

Agricultural nitrogen (N) inputs can accumulate as nitrate in the vadose zone, creating a source for subsequent leaching (Akbariyeh et al., 2019). Hydrological connectivity influences the pathways and timing of nitrate export, linking changes in water movement to the transport of stored N (Xiao et al., 2024; Wu et al., 2025). Groundwater level recovery can enhance nitrate leaching from the vadose zone and increase nitrate loading to the aquifer (Zang et al., 2022). The accompanying increase in soil saturation can also restrict oxygen transport and favor microbial nitrate reduction, with nitrate attenuation reported under particular groundwater level and substrate conditions (Li et al., 2023a; Zuo et al., 2023; Roumelis et al., 2025). The potential for both nitrate mobilization and concentration decline highlights the importance of soil biogeochemical conditions in determining nitrate persistence during wetting. Vadose zone sediments are vertically heterogeneous in carbon availability, indigenous N pools, texture and microbial composition, creating distinct initial conditions for N transformation (Li et al., 2023b; Chen et al., 2024). Increasing water saturation modifies these conditions by restricting oxygen diffusion and potentially favoring nitrate reduction before full saturation (Oh et al., 2023). Seasonal water-level fluctuations also produce contrasting patterns of microbial N transformation across soil textures and sampling depths (Zhang et al., 2023). These observations suggest that soils collected from different depths may exhibit different nitrate trajectories because their initial physical, chemical and microbial characteristics influence the conditions established during wetting. It remains unclear whether an equivalent increase in saturation produces comparable nitrate concentration decline under a common N input, and how the timing and extent of this decline vary among soils with contrasting initial properties.

Carbon availability can further modify nitrate transformation once oxygen supply becomes restricted. Heterotrophic nitrate reduction depends on suitable electron donors, and labile organic carbon can substantially influence nitrate persistence in deep vadose zone materials (Li et al., 2023b). The form of incoming N introduces additional differences. Ammonium can contribute to nitrate formation through nitrification, while readily biodegradable dissolved organic nitrogen (DON) can supply inorganic N through mineralization and carbon for heterotrophic metabolism (Liu et al., 2023; Wang and Li, 2024). Carbon and nitrate availability can also influence competition between denitrification and dissimilatory nitrate reduction to ammonium (DNRA), with different consequences for N loss and retention (Hao et al., 2025; Liao et al., 2025). These roles make it important to distinguish the influence of additional carbon under a common nitrate input from the concentration trajectories following different N substrates. Comparisons within the same soil and moisture setting can clarify how these substrate conditions influence nitrate accumulation and decline. Changes in groundwater levels and geochemical conditions can alter bacterial community composition and the functional capacity for N cycling (Zuo et al., 2023; Ren et al., 2026). Combining microbial information with hydrochemical and isotopic evidence helps place potential N pathways within their environmental context (Wang et al., 2024). DNA-based functional gene abundance characterizes the genetic potential present in a community, while gene expression and environmental conditions influence its activity (Yoshida et al., 2012). Chemical trajectories and functional gene abundances may therefore show different treatment patterns. A complementary question concerns whether bacterial community composition and N-cycling functional potential at the end of incubation vary among moisture and substrate treatments in association with the observed changes in dissolved N forms. Examining these measurements together can provide a more integrated view of nitrate persistence and the microbial capacities associated with sustained exposure to contrasting moisture and substrate conditions.

To address these gaps, this study examined how soil saturation and substrate availability influence nitrate dynamics and microbial N cycling potential in vadose-zone soils. Two complementary microcosm experiments used soils collected from three depths within a single profile in Beijing, China. Incubations at fixed levels of 50%, 80 and 100% water filled pore space (WFPS) compared nitrate concentration changes among the three soils under a common nitrate input. A subsequent experiment with the deepest soil at 100% WFPS evaluated nitrate, nitrate plus glucose, ammonium and glycine additions. The experiments examined the hypothesis that increasing saturation and labile carbon supply would enhance nitrate concentration decline, while substrate composition would influence the timing of changes among dissolved N forms. Chemical time series, together with bacterial community composition and endpoint functional gene abundance, provided complementary perspectives on nitrogen dynamics and microbial functional potential. These comparisons aimed to clarify the soil and substrate conditions associated with nitrate persistence under increased saturation, with implications for interpreting vadose-zone biogeochemical changes during groundwater recovery.

## 2 Materials and methods

### 2.1 Soil sampling and initial characterization

The incubation experiments were conducted in microcosms (Figure 1). Soil samples were collected from a greenhouse agricultural field in Suocao Village, Huairou District, Beijing, China, located in an area where groundwater levels have risen markedly in recent years. A single soil profile was excavated and divided into three sampling intervals: D1 at 1 ~ 2 m, D2 at 2 ~ 4 m and D3 at 4 ~ 6 m. After removing approximately 2 ~ 3 cm of disturbed material from the exposed profile, five subsamples were collected laterally from each interval and homogenized to form one composite sample. Thus, each depth was represented by one composite soil sample from the same profile. Visible roots, gravel and other debris were removed immediately after sampling. Fresh soils used for incubation were transported to the laboratory under cooled conditions and processed within 24 h. Subsamples for initial physicochemical characterization were air dried and passed through a 2 mm sieve where appropriate, while those used for microbial analysis were stored at −80 °C. Gravimetric water content, total nitrogen (TN), dissolved organic carbon (DOC), total dissolved nitrogen (TDN), dissolved organic nitrogen (DON), ammonium-N (NH_4_^+^-N), nitrite-N (NO_2_^−^-N), nitrate-N (NO_3_^−^-N) and pH were determined before incubation.

**Figure 1:**
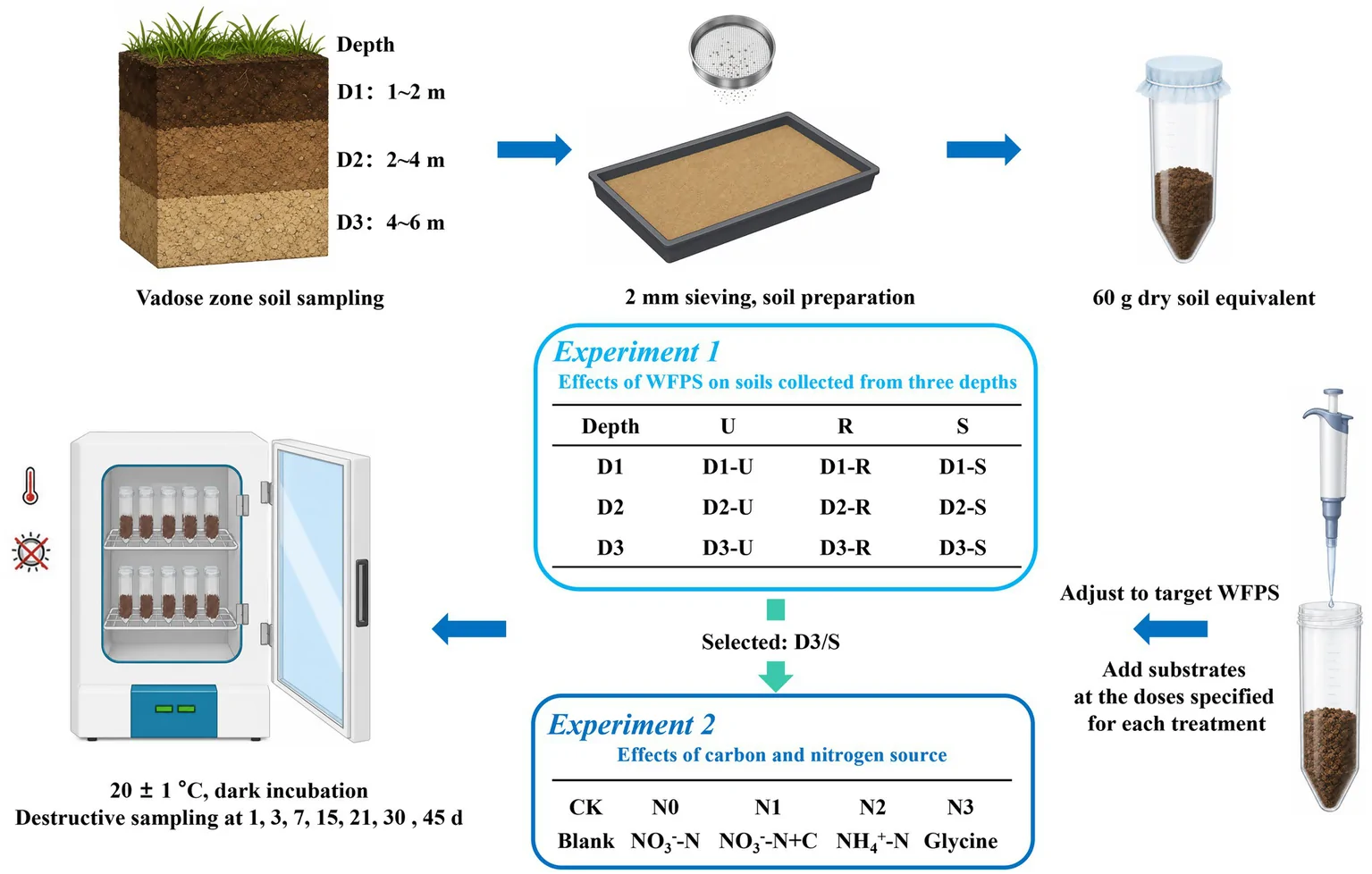
The incubation experiment design.

### 2.2 Microcosm incubation experiments

#### 2.2.1 Effects of WFPS on soils collected from three depths

Fresh soil equivalent to 60 g dry weight was placed in 100 mL polyethylene tubes and gently packed to approximate the original bulk density. The microcosms were preincubated in the dark at 20 ± 1\u00B0C for 7 days. Three levels of water filled pore space (WFPS), 50%, 80 and 100%, were established for each soil and are hereafter denoted U, R and S, respectively. These static WFPS treatments served as an experimental proxy for contrasting soil saturation levels that may accompany groundwater recovery. All tubes were covered with sterile gas permeable membranes. Soil moisture was maintained by daily weighing and replenishment with ultrapure water.

Following preincubation, NaNO_3_ was added to all microcosms at 30 mg N kg^−1^ dry soil. The experiment therefore comprised nine treatments: D1-U, D1-R, D1-S, D2-U, D2-R, D2-S, D3-U, D3-R and D3-S. Samples were destructively collected after 1, 3, 7, 15, 21, 30 and 45 days. Day 0 was defined as the day of substrate addition following the 7-day preincubation. Three independent microcosms were prepared for each treatment and sampling time.

#### 2.2.2 Effects of carbon and nitrogen source

The second incubation experiment was conducted using D3 soil at 100% WFPS. Five treatments were considered: CK without additional C or N; N0 supplied with NaNO_3_; N1 supplied with NaNO_3_ and glucose; N2 supplied with NH_4_Cl; and N3 supplied with glycine as a readily available organic N source. All treatments receiving external N were amended at 30 mg N kg^−1^ dry soil. Glucose supplied 300 mg C kg^−1^ in N1, corresponding to an added C/N mass ratio of 10. Addition of glycine supplied 30 mg N kg^−1^ together with 51.43 mg C kg^−1^. Incubation conditions and sampling times were identical to those described above. N0 was represented by D3-S. Physicochemical parameters were measured at all seven sampling times.

### 2.3 Physicochemical analyses

Soil water content was determined gravimetrically after drying at 105 °C. WFPS was calculated from volumetric water content and soil porosity:


$$\text{WFPS}=\frac{\theta_{v}}{n}\times 100\%$$ (1)

Where $\theta_{v}$ is volumetric water content and n is soil porosity. Volumetric water content was calculated from gravimetric water content and bulk density, and porosity was derived from soil bulk density and particle density.

For dissolved C and N analyses, 5.0 g fresh soil was extracted with 25 mL ultrapure water by shaking at 200 rpm for 24 h. The suspensions were centrifuged at 4000 rpm for 10 min and the supernatants were passed through 0.45 μm membranes. Dissolved N concentrations were expressed as mg N kg^−1^ dry soil, and DOC concentrations as mg C kg^−1^ dry soil. NO_3_^−^-N was measured using the ultraviolet dual wavelength method at 220 and 275 nm after addition of HCl and sulfamic acid. NO_2_^−^-N was determined using the sulfanilamide and N-(1-naphthyl) ethylenediamine colorimetric method at 540 nm, and NH_4_^+^-N was measured using the Nessler method at 420 nm. TDN was determined by ultraviolet spectrophotometry after persulfate oxidation, while DOC was measured using a total organic carbon analyzer. All concentrations were expressed on a dry soil mass basis. Soil pH was measured in a 1:2.5 soil to water suspension. Redox potential (Eh) was measured directly in fresh soil using a platinum combination electrode. DON was calculated as the difference between TDN and the sum of the measured inorganic N concentrations:


$$\mathrm{DON}=\mathrm{TDN}-NH_{4}^{+}-NO_{2}^{-}-NO_{3}^{-}$$ (2)

## 3 Microbial analyses

Microbial analyses were conducted on day 0 and day 45. The day 0 samples comprised D1, D2 and D3 following preincubation. The day-45 samples comprised the nine WFPS treatments together with CK, N1, N2 and N3; N0 was represented by D3-S. Three biological replicates were analyzed for each sample group. For both bacterial community and functional gene analyses, DNA was extracted separately from each biological replicate, and each replicate was sequenced independently; group mean abundances were calculated after processing the individual replicate datasets.

### 3.1 Bacterial community composition

Total microbial DNA was extracted using an E. Z. N. A.® Soil DNA Kit (Omega Bio-tek, Norcross, GA, United States). DNA quality was examined by 1% agarose gel electrophoresis and a NanoDrop 2000 spectrophotometer. The V3-V4 region of the bacterial 16S rRNA gene was amplified using primers 338F (ACTCCTACGGGAGGCAGCAG) and 806R (GGACTACHVGGGTWTCTAAT) and sequenced on an Illumina NextSeq 2000 platform according to the standard protocol of Majorbio Bio-Pharm Technology Co., Ltd. (Shanghai, China). After demultiplexing, sequences were quality filtered using fastp and paired reads were merged using FLASH. High quality sequences were denoised and chimeras were removed using DADA2 within QIIME2, generating amplicon sequence variants (ASVs) (Callahan et al., 2016; Bolyen et al., 2019). Taxonomic assignment was performed against the SILVA 16S rRNA database version 138.2, and community composition was evaluated at the phylum and genus levels.

### 3.2 Absolute abundance of nitrogen cycling functional genes

Total genomic DNA was extracted using a Mag-Bind® Soil DNA Kit (Omega Bio-tek, Norcross, GA, United States). For absolute quantification, 10 Spike in DNA sequences with known copy numbers across five concentration gradients were added to each DNA extract, accounting for approximately 3% of the extracted DNA. DNA was fragmented to approximately 350 bp, libraries were prepared using the NEXTFLEX Rapid DNA-Seq Kit, and shotgun metagenomic sequencing was performed on a DNBSEQ-T7 platform with paired end 150 bp reads. Raw reads were quality filtered using fastp. Spike in sequences were mapped to the metagenomic reads using Bowtie2, and a standard curve was constructed for each sample from the relationship between their known copy numbers and length normalized read counts. After removal of Spike in reads, the remaining sequences were assembled using MEGAHIT. Open reading frames were predicted using Prodigal, and a nonredundant gene catalog was constructed using CD-HIT at 90% sequence identity and 90% coverage. Reads from individual samples were mapped to the gene catalog, and absolute gene copy numbers were inferred from the corresponding Spike in standard curves and normalized to soil mass. Functional annotation was performed against the KEGG database using DIAMOND with an E value threshold of 1 × 10^−5^ (Buchfink et al., 2015). Genes associated with nitrification, denitrification, and DNRA were identified from their KEGG Orthology annotations. Absolute gene abundances, expressed as copies g^−1^ soil, characterized nitrogen-cycling functional potential on days 0 and 45.

### 3.3 Calculations and data analysis

Unless otherwise specified, concentration decreases were calculated between incubation days 1 and 45. The absolute decrease was calculated as $C_{1}-C_{45}$ , where $C_{1}$ and $C_{45}$ denote the corresponding concentrations on days 1 and 45. Negative values indicate a net concentration increase over this interval. Incubation measurements were summarized as means ± standard errors (SE), with three independent microcosms per treatment and sampling time. SEs of concentration decreases, percentage decreases and changes in treatment-control differences were estimated by first-order error propagation from the corresponding concentration means, assuming independence among microcosms sampled at different times and under different treatments.

Because measurable changes in N also occurred in CK, differences relative to the control were calculated for the nitrogen source experiment as:


$$\Delta C_{i,t}=C_{i,t}-C_{\mathrm{CK},t}$$ (3)

Where $C_{i,t}$ and $C_{\mathrm{CK},t}$ are the concentrations of a given N form in treatment i and CK at time t , respectively. For the substrate experiment, the percentage decrease in the concentration difference relative to CK was calculated as:


$$R_{i}=\frac{\Delta C_{i,1}-\Delta C_{i,45}}{\Delta C_{i,1}}\times 100\%$$ (4)

To compare temporal patterns, the incubation period was divided into days 1 ~ 15, 15 ~ 30 and 30 ~ 45. The intervals from days 1 ~ 15, 15 ~ 30 and 30 ~ 45 represented elapsed durations of 14, 15 and 15 days, respectively. The average decrease rate during each interval was calculated as:


$$v_{t_{1}-t_{2}}=\frac{C_{t_{1}}-C_{t_{2}}}{t_{2}-t_{1}}$$ (5)

And stage contributions were expressed as percentages of the total decrease between days 1 and 45:


$$P_{t_{1}-t_{2}}=\frac{C_{t_{1}}-C_{t_{2}}}{C_{1}-C_{45}}\times 100\%$$ (6)

Concentration profiles showing an initial decrease followed by an apparent residual level were fitted using a first order decay model with a nonzero asymptote:


$$C_{t}=C_{r}+(C_{0}-C_{r})e^{-\mathrm{kt}}$$ (7)

Where $C_{t}$ is the concentration at time t , $C_{0}$ is the model-estimated concentration on day 0, $C_{r}$ is the fitted asymptotic concentration and k is the apparent decline coefficient. The fitted declining fraction was calculated as:


$$f_{r}=\frac{C_{0}-C_{r}}{C_{0}}\times 100\%$$ (8)

And its apparent half life was calculated from:


$$t_{1/2}=\frac{\ln2}{k}$$ (9)

## 4 Results

### 4.1 Saturation and nitrate dynamics in soils collected from three depths

The three vadose zone soils differed substantially in their initial C and N status (Table 1). D1 contained the highest concentrations of TN, NO_3_^−^-N and TDN, whereas D2 had the lowest DOC and DON contents. D3 had the highest initial DOC concentration, at 66.43 mg C kg^−1^ approximately 1.4 and 1.6 times the concentrations in D1 and D2, respectively. After 30 mg N kg^−1^ nitrate was added, NO_3_^−^-N accounted for 72.13 ~ 80.50% of TDN on day 1 and became the dominant form of dissolved N in all treatments (Figure 2).

**Table 1:** Initial physicochemical properties of vadose zone soils at three depths.

Property	D1	D2	D3
Depth (m)	1 ~ 2	2 ~ 4	4 ~ 6
Water content (%)	9.83 ± 0.15	12.16 ± 0.23	17.39 ± 0.32
TN (g N kg^−1^)	0.83 ± 0.02	0.25 ± 0.01	0.41 ± 0.01
DOC (mg C kg^−1^)	47.64 ± 0.68	41.01 ± 0.75	66.43 ± 0.85
NO_3_^−^-N (mg N kg^−1^)	9.76 ± 0.19	2.56 ± 0.06	5.96 ± 0.14
NH_4_^+^-N (mg N kg^−1^)	1.39 ± 0.04	1.16 ± 0.02	1.44 ± 0.03
TDN (mg N kg^−1^)	24.20 ± 0.56	10.60 ± 0.25	18.30 ± 0.40
DON (mg N kg^−1^)	12.97 ± 0.25	6.83 ± 0.13	10.83 ± 0.26
pH	8.35 ± 0.19	8.58 ± 0.21	8.26 ± 0.19
Bulk density (g cm^−3^)	1.41 ± 0.07	1.37 ± 0.02	1.43 ± 0.03

Values are means ± standard errors (SE) of three determinations per composite soil sample (*n* = 3).

**Figure 2:**
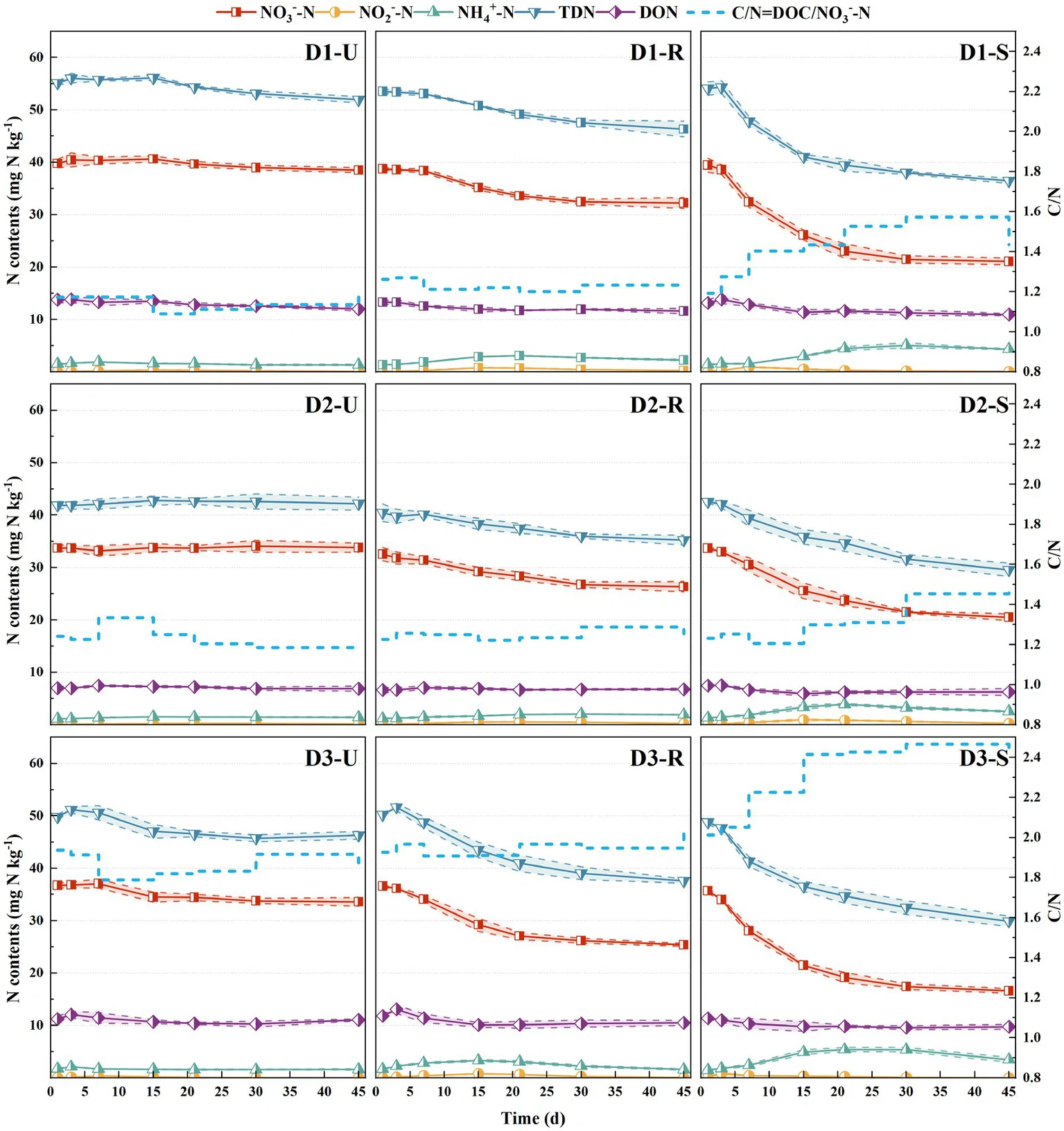
Temporal changes in dissolved nitrogen pools at different soil depths and WFPS levels. Shaded areas show the mass ratio of DOC to NO_3_^−^-N, labeled C/N and plotted against the right-hand axis. Symbols represent treatment means, and error bars indicate standard errors from three independent microcosms per sampling time.

NO_3_^−^-N concentrations remained relatively stable at 50% WFPS: between days 1 and 45, D1-U and D3-U showed declines of 3.15 and 8.57%, respectively, whereas D2-U showed a slight increase of 0.18% (Figure 2, Table 2). At 80% WFPS, the corresponding declines were 16.79%, 19.10 and 30.60% in D1-R, D2-R and D3-R, respectively (Figure 2). At 100% WFPS, NO_3_^−^-N declined by 46.53%, 39.15 and 53.52% in D1-S, D2-S and D3-S, respectively, relative to their concentrations on day 1 (Figure 2). Although D1-S and D3-S showed similar absolute decreases of 18.36 and 19.12 mg N kg^−1^, respectively, their day 1 concentrations differed: 39.45 mg N kg^−1^ in D1-S and 35.72 mg N kg^−1^ in D3-S. TDN declined by 30.56 ~ 38.78% between days 1 and 45 across the three 100% WFPS treatments (Table 2). NO_2_^−^-N accumulated only transiently and remained below 1 mg N kg^−1^ throughout the experiment. The 100% WFPS treatments reached higher NH_4_^+^-N maxima (3.88 ~ 5.42 mg N kg^−1^) than the 50% WFPS treatments (1.52 ~ 2.12 mg N kg^−1^; Figure 2). Although DON accounted for a larger fraction of TDN at the end of incubation in D1-S and D3-S, its absolute concentration had declined. The higher proportional contribution mainly reflected the more rapid decrease in nitrate rather than an accumulation of DON.

**Table 2:** Changes in nitrate, TDN and DOC and minimum Eh under different WFPS conditions.

Sample	WFPS %	NO_3_^−^-N decrease mg N kg^−1^	NO_3_^−^-N decrease %	TDN decrease %	DOC decrease %	Min Eh mV	Day of min Eh
D1-U	50	1.25 ± 0.54	3.15 ± 1.31	5.77 ± 0.70	3.18 ± 0.36	342 ± 5.9	21
D1-R	80	6.50 ± 0.62	16.79 ± 1.59	13.44 ± 1.66	19.87 ± 1.73	173 ± 10.9	45
D1-S	100	18.36 ± 0.85	46.53 ± 1.39	32.60 ± 1.05	35.58 ± 1.61	58 ± 4.1	21
D2-U	50	−0.06 ± 0.61	−0.18 ± 1.82	−0.71 ± 1.90	3.27 ± 2.85	335 ± 4.5	30
D2-R	80	6.22 ± 0.92	19.10 ± 2.50	12.94 ± 2.50	17.24 ± 2.06	181 ± 4.1	30
D2-S	100	13.20 ± 0.40	39.15 ± 1.13	30.56 ± 1.74	27.51 ± 2.99	92.0 ± 13.0	30
D3-U	50	3.15 ± 0.55	8.57 ± 1.46	7.03 ± 0.97	11.50 ± 1.18	300 ± 1.8	21
D3-R	80	11.20 ± 0.19	30.60 ± 0.47	25.19 ± 0.46	27.15 ± 2.22	118 ± 6.3	21
D3-S	100	19.12 ± 0.36	53.52 ± 0.77	38.78 ± 1.24	44.32 ± 2.16	39.0 ± 3.6	15

Concentration changes are estimates ± propagated SE, calculated from treatment means based on three independent microcosms per sampling time (*n* = 3). Absolute decreases were calculated as day-1 minus day-45 concentrations, and percentage decreases were expressed relative to the corresponding day 1 concentrations; negative values indicate concentration increases.

Higher WFPS treatments showed larger DOC declines and lower Eh minima (Supplementary Figures S1a,b; Table 2). At 100% WFPS, DOC decreased by 35.58% in D1-S and 27.51% in D2-S, while the decrease in D3-S reached 44.32% between days 1 and 45 (Supplementary Figure S1b). The minimum Eh ranged from 39 to 92 mV at 100% WFPS, compared with 300 ~ 342 mV at 50% WFPS (Supplementary Figure S1a). D3-S reached its minimum Eh on day 15, earlier than D1-S and D2-S, which reached their minima on days 21 and 30. By comparison, pH changed only slightly during incubation and remained within the weakly alkaline range of 8.07 ~ 8.60.

At 100% WFPS, 61.63–74.50% of the total NO_3_^−^-N decline between days 1 and 45 occurred between days 1 and 15, compared with 53.57 ~ 65.73% at 80% WFPS (Table 3). The average rates between days 1 and 15 were 0.953 mg N kg^−1^ d^−1^ in D1-S, 0.581 mg N kg^−1^ d^−1^ in D2-S and 1.017 mg N kg^−1^ d^−1^ in D3-S. After day 30, nitrate changed little in all three saturated treatments. For each soil, the apparent decay coefficient was higher at 100% than at 80% WFPS. At 100% WFPS, the coefficients ranged from 0.0567 to 0.0890 d^−1^, and the fitted declining fractions ranged from 44.89 to 56.30% (Table 3). Given its greater nitrate decrease, higher early average decline rate and larger fitted declining fraction, D3 at 100% WFPS was selected for the subsequent experiment.

**Table 3:** Temporal characteristics and empirical model parameters of nitrate concentration decline at 80 and 100% WFPS.

Sample	Mean decrease rate during 1 ~ 15 d (mg N kg^−1^ d^−1^)	Contribution of days 1 ~ 15 to total decrease (%)	*k* (d^−1^)	Fitted declining fraction (%)	Apparent half life (d)
D1-R	0.255	54.97	0.0463	22.06	14.97
D1-S	0.953	72.67	0.0857	50.55	8.09
D2-R	0.238	53.57	0.0407	24.05	17.03
D2-S	0.581	61.63	0.0567	44.89	12.22
D3-R	0.526	65.73	0.0651	35.05	10.65
D3-S	1.017	74.50	0.0890	56.30	7.79

R and S denote 80 and 100% WFPS, respectively. Stage metrics were calculated from treatment mean concentrations (*n* = 3 independent microcosms per sampling time).

### 4.2 Nitrogen dynamics under different substrate additions at 100% WFPS

On day 1, NO_3_^−^-N dominated the dissolved N pool in N0 and N1, NH_4_^+^-N in N2, and DON in CK and N3 (Figure 3a). In CK, between days 1 and 45, NO_3_^−^-N decreased from 6.12 to 4.92 mg N kg^−1^, DON declined by 0.81 mg N kg^−1^, and NH_4_^+^-N increased by 0.61 mg N kg^−1^ (Figure 3b).

**Figure 3:**
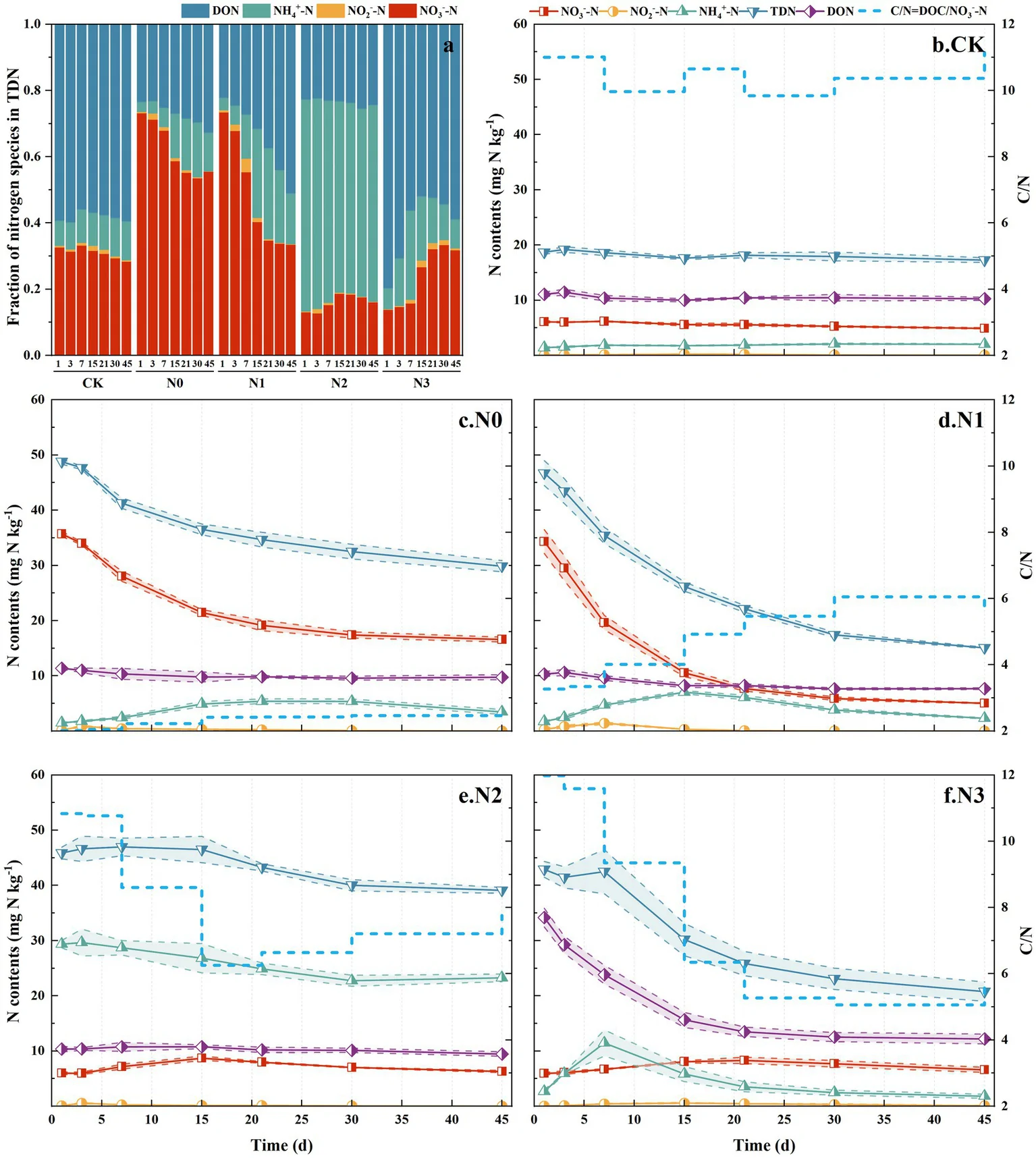
Composition and temporal changes of dissolved nitrogen under different substrate treatments. **(a)** Relative contributions of NO_3_^−^-N, NO_2_^−^-N, NH_4_^+^-N and DON to TDN. **(b–f)** Concentration trajectories in CK, N0, N1, N2 and N3, respectively.

When nitrate was added without an additional organic C source, NO_3_^−^-N in N0 decreased from 35.72 to 16.60 mg N kg^−1^ between days 1 and 45, corresponding to a decline of 53.52% (Figure 3c). TDN decreased concurrently from 48.81 to 29.88 mg N kg^−1^. With glucose addition, NO_3_^−^-N in N1 decreased from 34.35 mg N kg^−1^ on day 1 to 5.05 mg N kg^−1^ by day 45, representing a decline of 85.30% (Figure 3d). TDN simultaneously fell from 46.75 to 15.07 mg N kg^−1^. Both NO_2_^−^-N and NH_4_^+^-N reached higher transient maxima than those observed in N0, attaining 1.441 and 7.047 mg N kg^−1^ on days 7 and 15, respectively (Figures 3c,d). NH_4_^+^-N in N2 remained close to 29 mg N kg^−1^ during the first 3 days and subsequently decreased to 23.28 mg N kg^−1^ by day 45. NO_3_^−^-N increased from 6.02 mg N kg^−1^ on day 1 to a maximum of 8.72 mg N kg^−1^ on day 15, and then decreased during the remainder of the incubation. Between days 1 and 45, TDN declined from 45.90 to 39.13 mg N kg^−1^ (Figure 3e). Following glycine addition, DON in N3 decreased from 34.20 to 12.22 mg N kg^−1^ between days 1 and 45. NH_4_^+^-N reached its maximum first, on day 7, followed by NO_2_^−^-N on day 15 and NO_3_^−^-N on day 21. The fraction of TDN represented by DON decreased from 79.64% on day 1 to 58.76% on day 45 (Figures 3a,f).

At each sampling time, the concentration difference was calculated as the amended treatment minus CK. Relative to the corresponding day 1 differences, these differences declined by 60.54% for NO_3_^−^-N in N0, 99.54% for NO_3_^−^-N in N1, 24.02% for NH_4_^+^-N in N2 and 91.59% for DON in N3 by day 45 (Table 4). In N1, NO_3_^−^-N exceeded CK by 28.23 mg N kg^−1^ on day 1 and by only 0.13 mg N kg^−1^ on day 45. These comparisons describe changes in concentration differences and do not separately track native and added N. For the concentration differences relative to CK, days 1–15 accounted for 76.56%, 82.75 and 82.42% of the total day 1 ~ 45 declines in N0, N1 and N3, respectively (Table 4). During this period, the average decline rates of the NO_3_^−^-N differences were 0.980 mg N kg^−1^ d^−1^ in N0 and 1.661 mg N kg^−1^ d^−1^ in N1. Unlike nitrate in N0 and N1, the main decrease in NH_4_^+^-N in N2 relative to the control occurred between days 15 and 30, followed by a slight increase between days 30 and 45. Addition of glucose increased the apparent decay coefficient from 0.0890 to 0.1147 d^−1^ and expanded the fitted declining fraction from 56.30 to 86.39%. The corresponding apparent half-lives were 7.79 days in N0 and 6.04 days in N1 (Table 4).

**Table 4:** Concentration changes and empirical kinetic characteristics under different substrate treatments.

A. Concentration changes between days 1 and 45 and minimum mean Eh						
Sample	Target N form	Concentration decrease (%)	Decrease in treatment–CK difference (%)	DOC decrease (%)	Min mean Eh (mV)	Day of min Eh
N0	NO_3_^−^-N	53.52 ± 0.77	60.54 ± 0.95	44.32 ± 2.16	39 ± 3.6	15
N1	NO_3_^−^-N	85.30 ± 0.54	99.54 ± 0.24	74.08 ± 1.09	−89 ± 12.0	15
N2	NH_4_^+^-N	20.78 ± 1.69	24.02 ± 1.76	24.90 ± 2.25	79 ± 1.2	21
N3	DON	64.27 ± 1.81	91.59 ± 2.35	48.30 ± 1.62	68 ± 9.2	21

B. Main decline periods and empirical model parameters					
Sample	Main decline period	Contribution to total decrease in treatment–CK difference (%)	*k* (d^−1^)	Fitted declining fraction (%)	Apparent half-life (d)
N0	1 ~ 15	76.56	0.0890	56.30	7.79
N1	1 ~ 15	82.75	0.1147	86.39	6.04
N2	15 ~ 30	65.46	0.0391	30.13	17.73
N3	1 ~ 15	82.42	0.1153	65.84	6.01

Changes in panel A are estimates ± propagated SE, based on three independent microcosms per treatment and sampling time. Minimum mean Eh and its SE refer to the indicated sampling day. The main decline period is the interval contributing the largest decrease in the treatment–CK concentration difference between days 1 and 45. Stage contributions in panel B were calculated from these differences. Kinetic parameters describe fits to the unadjusted concentrations of the target N form. The apparent half-life applies to the fitted declining component.

The differences among N treatments were accompanied by distinct changes in DOC and Eh (Supplementary Figures S2a,b). Relative to day 1, DOC declined by 74.08% in N1 by day 45, compared with 44.32% in N0, 24.90% in N2 and 48.30% in N3 (Supplementary Figure S2b; Table 4). N1 reached the lowest Eh, at −89 mV, compared with a minimum of 39 mV in N0 (Supplementary Figure S2a). pH ranged from 7.83 to 8.37 across the substrate treatments, with the minimum in N1 on day 15 (Supplementary Figure S2a).

### 4.3 Changes in microbial community composition

Bacterial community composition was compared between the initial soils and the day-45 samples. Acidobacteriota, Pseudomonadota, Bacillota, Actinomycetota and Chloroflexota constituted the dominant phyla in most samples (Supplementary Figures S3a,c). Bacillota showed higher relative abundances on day 45 than in the initial samples of all three soils. Its day-45 relative abundance ranged from 17.58 to 21.91% in D1 and from 6.23 to 9.49% in D2. In D3, its relative abundance increased from 3.33% initially to 14.14 ~ 14.55% across the WFPS treatments. Actinomycetota and Gemmatimonadota generally showed lower relative abundances in the day-45 samples of D2 and D3 than in their corresponding initial soils (Supplementary Figure S3a). Differences among the soils were also evident at the genus level. In D1, the relative abundance of *Peribacillus* increased from 3.13% initially to 4.36 ~ 5.60% across the WFPS treatments on day 45. In D2, *Nitrospira* reached its highest relative abundance under 80% WFPS, accounting for 3.35% of the community. The relative abundance of *Paenibacillus* in D3 increased from 0.22% initially to 1.24 ~ 1.59% on day 45 (Supplementary Figure S3b).

Among the D3 substrate treatments, N0 and N2 had dominant phylum profiles broadly similar to CK on day 45. Bacillota accounted for 37.55% of the community in N1 compared with 14.94% in CK. Over the same comparison, Acidobacteriota accounted for 11.47% in N1 and 20.90% in CK. N3 showed a different composition, with Pseudomonadota accounting for 29.15% of the community compared with 16.64% in CK (Supplementary Figure S3c). At the genus level, *Clostridium* and *Thermincola* accounted for 11.00 and 8.88% of the N1 community, respectively, compared with 0.51 and 0.21% in CK. In N3, *Aromatoleum*, *Ammoniphilus* and *Pseudomonas* accounted for 12.07%, 3.87 and 3.09%, respectively. *Nitrospira* also differed among the substrate treatments, representing 3.09% of the community in N3, 2.34% in CK and 1.18% in N1 (Supplementary Figure S3d).

### 4.4 Nitrogen cycling functional gene abundances

Genes assigned to nitrification, denitrification and DNRA were detected in all original soils and incubation treatments. The treatment comparisons below refer to gene abundances measured on day 45, with the initial soils providing the reference for changes during incubation.

In D1, most genes associated with N cycling were more abundant under 50% WFPS than under the two wetter conditions (Figure 4). A broadly similar pattern was observed in D2. D3 differed from the shallower soils, with many genes associated with nitrate reduction reaching their highest abundance at 80% WFPS. The combined abundance of genes assigned to denitrification was approximately 1.50 × 10^7^ copies g^−1^ in the original D3 soil. On day 45, the corresponding abundances were 2.97 × 10^7^, 4.49 × 10^7^ and 2.21 × 10^7^ copies g^−1^ in D3-U, D3-R and D3-S, respectively (Figure 4h). Genes assigned to DNRA showed a comparable pattern, with the highest combined abundance on day 45 in D3-R (Figure 4i). *nirK* increased from 2.89 × 10^6^ to 4.70 × 10^6^ copies g^−1^, while *nosZ* increased from 1.62 × 10^6^ to 2.52 × 10^6^ copies g^−1^ between the initial D3 soil and D3-S on day 45.

**Figure 4:**
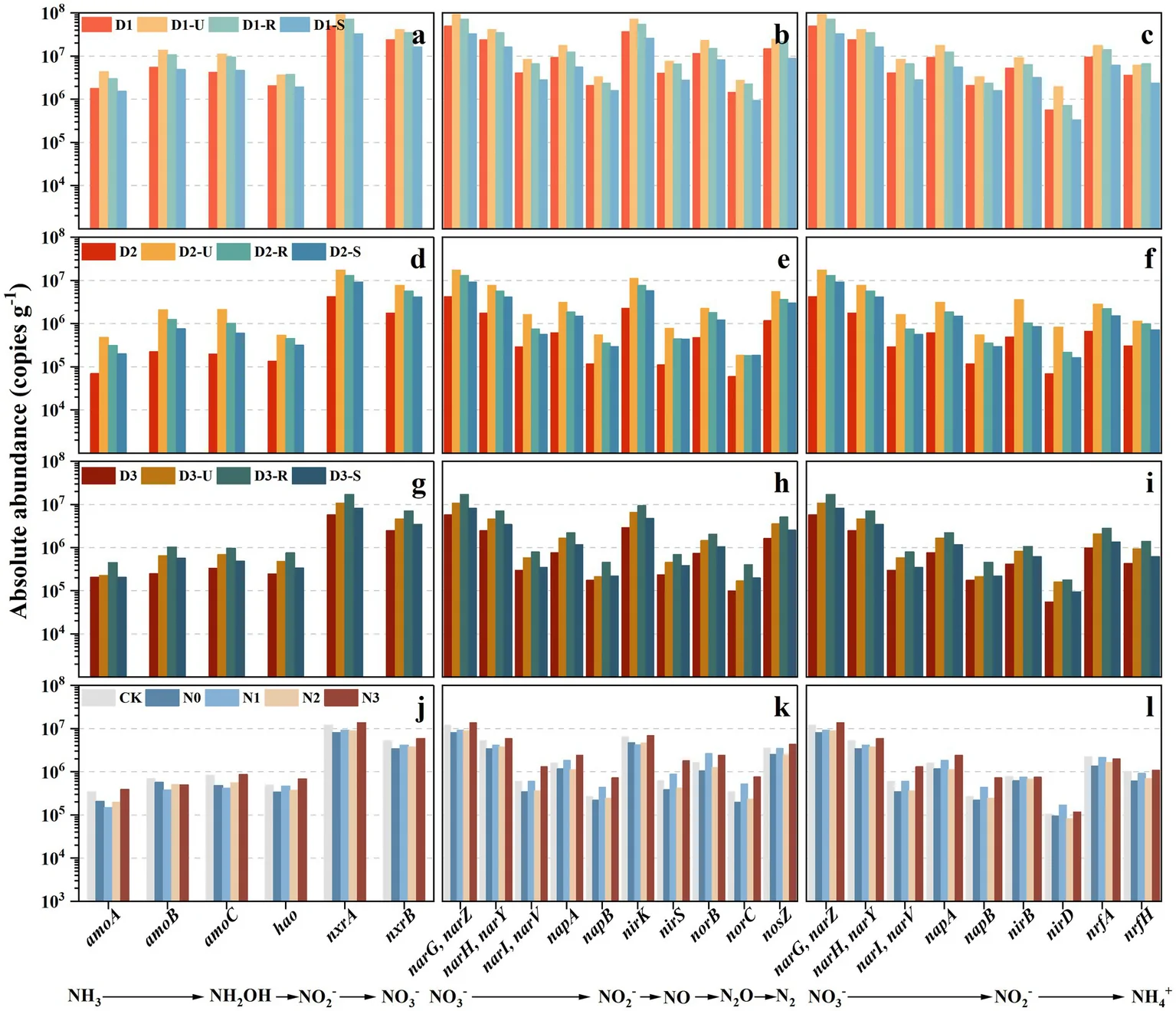
Absolute abundances of nitrogen cycling functional genes under variable saturation and substrate conditions. Panels **(a–i)** represent D1, D2, and D3 soils under different WFPS levels, including nitrification **(a,d,g)**, denitrification **(b,e,h)**, and DNRA **(c,f,i)**, respectively. Panels **(j–l)** represent different nitrogen substrate treatments for nitrification **(j)**, denitrification **(k)**, and DNRA **(l)**. Functional genes were classified according to KEGG Orthology annotations.

Nitrate addition alone (N0) did not increase the total abundance of genes assigned to denitrification relative to CK. Relative to CK, N1 showed higher abundances of some genes assigned to intermediate reduction steps, without a uniform increase across all genes assigned to denitrification. The abundance of *nirS* increased from 6.14 × 10^5^ copies g^−1^ in CK to 8.79 × 10^5^ copies g^−1^ in N1. Over the same comparison, *norB* increased from 1.64 × 10^6^ to 2.63 × 10^6^ copies g^−1^, and *norC* from 3.42 × 10^5^ to 5.20 × 10^5^ copies g^−1^ (Figure 4k). In contrast, *nosZ* remained close to the abundance measured in CK. In N2, the combined abundance of genes assigned to ammonia oxidation (*amoA*, *amoB*, *amoC* and *hao*) was approximately 73% of the CK value on day 45 (Figure 4j). In N3, relative to CK, the combined abundance of genes assigned to denitrification increased by approximately 24%, while those assigned to DNRA and nitrification increased by approximately 17 and 12%, respectively (Figures 4j–l).

The treatment rankings for endpoint gene abundances differed from those for NO_3_^−^-N decline between days 1 and 45. D3-R contained a larger overall abundance of genes associated with nitrate reduction than D3-S, although nitrate decreased to a greater extent in D3-S (Figures 2, 4h,i). Similarly, NO_3_^−^-N differed by only 0.13 mg N kg^−1^ between N1 and CK on day 45, whereas genes assigned to denitrification did not show uniformly higher abundances in N1 than in CK (Figure 4k).

Across the day-0 and day-45 samples from D1, D2 and D3, the abundances of the 10 most abundant nitrogen-cycling gene annotations in the saturation dataset correlated positively with NO_3_^−^-N, TDN and DON. Spearman’s ρ ranged from 0.682 to 0.717 for NO_3_^−^-N and from 0.692 to 0.736 for TDN, with all *p* < 0.01. For DON, ρ ranged from 0.494 to 0.692, with all *p* < 0.05. Correlations with DOC and Eh were weaker, with all *p* > 0.05 (Supplementary Figure S4; Supplementary Table S5). The D3 dataset, comprising the day 0 soil and the substrate treatments on day 45, showed a different pattern. The abundances of the 10 most abundant nitrogen-cycling gene annotations correlated positively with NO_2_^−^-N, with ρ ranging from 0.708 to 0.976 and all *p* < 0.05. The strongest correlations occurred for *nirA* and *nirK*, with ρ values of 0.976 and 0.952, respectively, and both *p* < 0.001. Correlations with DOC and Eh were negative across all 10 annotations, but only those involving *norB* reached *p* < 0.05, with ρ = −0.714 and *p* = 0.047 for each variable. For NO_3_^−^-N, all 10 correlations had *p* > 0.05 (Supplementary Figure S4b).

## 5 Discussion

### 5.1 Saturation and initial soil properties influence nitrate persistence

Water saturation strongly influenced nitrate persistence in the sampled soils. NO_3_^−^-N changed little at 50% WFPS but declined progressively more at 80 and 100% WFPS, alongside lower Eh (Figures 2; Supplementary Figure S1a). Greater water occupancy restricts oxygen diffusion through soil pores, potentially shifting microbial metabolism toward the use of alternative electron acceptors, including nitrate. The concurrent nitrate and Eh decreases therefore suggest that greater oxygen limitation contributed to the more extensive nitrate concentration decline under high WFPS. Comparable relationships between saturation, oxygen availability and nitrate reduction occur across soils with different pore structures (Mekala and Nambi, 2017; Oh et al., 2023).

The three soils entered incubation with contrasting carbon and nitrogen resources. D1 contained the largest TN and dissolved N pools, D2 had comparatively low DOC and DON concentrations, and D3 contained the most DOC (Table 1). Despite its larger DOC pool, D3 showed only a small nitrate concentration decrease at 50% WFPS. Higher saturation brought a different pattern: greater DOC decline accompanied lower Eh and a larger proportional nitrate decrease. This contrast suggests that the influence of organic carbon depended on the surrounding redox conditions. Carbon can supply both energy and reducing equivalents for microbial N cycling, while its utilization can increase oxygen demand. A comparatively large DOC pool, together with the earlier development of low Eh, thus offers a plausible explanation for the pronounced nitrate concentration decline in D3 under high WFPS. Carbon-supported nitrate reduction in subsoils similarly depends on the availability of suitable substrates and environmental conditions (Jahangir et al., 2012).

Starting nitrate concentrations also influenced the contrasts among soils. D1-S and D3-S showed similar absolute nitrate decreases between days 1 and 45, although D3-S had the larger proportional decrease; D2-S showed the smallest absolute decrease at 100% WFPS (Table 2). The variation among these soils therefore reflects their initial substrate conditions as well as the redox environment that developed during incubation. Carbon availability and soil texture can similarly help explain contrasting N distributions and possible transformation processes in deep vadose-zone profiles (Li et al., 2023b). Differences in initial pH, organic N availability and microbial composition may also influence substrate turnover. Physical properties, including bulk density and particle-size distribution, can modify pore connectivity and oxygen transport at the same WFPS. These considerations place the observed contrasts within the combined chemical and physical characteristics of the sampled soils.

The concentration trajectories also reveal when these differences developed. Much of the nitrate decline in treatments showing substantial decreases occurred between days 1 and 15, followed by slower changes later in incubation. Higher fitted decline coefficients and larger fitted declining fractions under higher WFPS and glucose addition capture changes in both the pace and extent of the decrease (Tables 3, 4). The concurrent decline in DOC suggests that changing carbon availability may have contributed to the later slowdown. Saturation and substrate supply consequently influenced both the size of the nitrate decrease and the period over which it developed.

### 5.2 Nitrogen inputs produce contrasting dissolved N trajectories

Glucose addition distinguished the nitrate dynamics of N1 from those of N0 despite their identical nitrate inputs. Between days 1 and 45, NO_3_^−^-N concentrations declined by 85.30% in N1 and 53.52% in N0 relative to their respective day 1 concentrations (Figures 3c,d). These changes coincided with a greater DOC decline and a much lower minimum Eh in N1, which reached −89 mV compared with 39 mV in N0 (Supplementary Figure S2). Readily available carbon supplies substrates for microbial metabolism, while increased respiratory demand can restrict oxygen availability within soil microsites. Together, these effects can favor nitrate reduction under high water saturation (Endress et al., 2024; Oh et al., 2026). The larger nitrate concentration decline following glucose addition therefore supports carbon availability as an important control on nitrate persistence within a common soil and moisture setting. Most of the decline occurred between days 1 and 15, indicating that the influence of substrate addition was strongest early in incubation, when both nitrate and readily available carbon were comparatively abundant.

Ammonium addition produced a different pattern. NH_4_^+^-N remained the dominant dissolved N form and decreased gradually, while NO_2_^−^-N accumulated transiently and NO_3_^−^-N increased until day 15 before subsequently declining (Figure 3e). This temporal behavior is consistent with limited ammonium oxidation during the early incubation period. Restricted oxygen transport at high water content provides a plausible explanation for the persistence of NH_4_^+^-N alongside transient nitrate accumulation, because moisture conditions influence the relative importance of oxidative and reductive N processes (Mekala and Nambi, 2017). By day 45, NH_4_^+^-N had declined by only 20.78% relative to day 1 and remained at 23.28 mg N kg^−1^. The largest decrease in the NH_4_^+^-N concentration difference relative to CK occurred during days 15 ~ 30, later than the main nitrate decline in N0 and N1 (Table 4). This delayed change highlights the comparatively prolonged persistence of ammonium under the same saturation conditions.

Glycine addition generated a clearer sequence of changes among organic and inorganic N forms. Rapid DON decline accompanied an NH_4_^+^-N maximum on day 7, followed by NO_2_^−^-N and NO_3_^−^-N maxima on days 15 and 21, respectively (Figure 3f). Amino acid mineralization followed by inorganic N turnover offers a plausible explanation for this sequence. Biodegradable DON can supply ammonium and subsequently contribute to nitrate formation in vadose-zone soils (Jones and Kielland, 2012; Liu et al., 2023). The later nitrate maximum in N3 than in N2 is compatible with the additional turnover of organic N preceding inorganic N accumulation. Continued nitrate accumulation through day 21, despite earlier DON decline, also suggests that precursor availability can influence nitrate dynamics beyond the period of most rapid change in the initial dominant N pool. Glycine differs from the other substrates because it supplies both C and N. At the applied N dose, glycine supplied 51.43 mg C kg^−1^, compared with the 300 mg C kg^−1^ supplied as glucose in N1. N3 therefore combined an organic N input with a smaller addition of carbon of a different chemical form. Mineralization can release inorganic N, while microbial use of the accompanying carbon can modify the conditions for subsequent N turnover. Direct uptake of glycine can also incorporate its carbon and nitrogen into microbial biomass (Xue et al., 2022). These alternative routes help explain how organic N additions can favor nitrate formation during one period and conditions conducive to nitrate decline during another (Liu et al., 2023). The nitrate maximum on day 21 followed by declining concentrations illustrates the resulting temporal complexity, which reflects the combined availability of carbon and different N substrates.

TDN provides additional context for these contrasting trajectories. In N3, TDN remained close to 43 mg N kg^−1^ between days 1 and 7, while DON decreased and the summed inorganic N concentration increased. N2 likewise maintained TDN near 46 ~ 47 mg N kg^−1^ through day 15 despite changes in NH_4_^+^-N and NO_3_^−^-N. These patterns distinguish early changes in the composition of the dissolved N pool from its subsequent decline in size (Figures 3e,f). In the nitrate treatments, declining TDN accompanied nitrate concentration decline, while temporary increases in NH_4_^+^-N and NO_2_^−^-N showed that other N forms also changed during incubation (Figures 3c,d). External inputs can additionally influence indigenous soil N turnover through changes in microbial demand and gross mineralization (Xu et al., 2023). Substrate availability thus influenced both the sequence of changes among N forms and the timing of decreases in the measured dissolved N pool.

### 5.3 Microbial community composition and nitrogen cycling potential

The stronger community changes following glucose addition connect carbon availability with the microbial environment in which nitrate decline occurred. Bacillota occupied a larger proportion of the N1 community than in CK, with particularly pronounced relative increases in *Clostridium* and *Thermincola* (Supplementary Figures S3c,d). These changes accompanied greater DOC decline and the lowest Eh among the substrate treatments. Abundant labile carbon can increase oxygen demand and favor anaerobic carbon metabolism, including fermentation, in Bacillota-dominated soil communities (Endress et al., 2024). Higher moisture has also coincided with greater relative abundances of Bacillota and *Clostridium* during organic matter decomposition (Lu et al., 2023). Such carbon processing offers a plausible link between substrate addition and the development of reducing conditions. Fermentative metabolism could additionally change the organic compounds available to nitrate-reducing populations, connecting carbon turnover with N cycling at the community level.

The functional gene profile in N1 showed differences among individual reduction steps. Relative to CK, *nirS*, *norB* and *norC* were more abundant, whereas *nosZ* remained comparatively stable (Figure 4k). These genes represent nitrite reduction, nitric oxide reduction and nitrous oxide reduction, respectively. Their contrasting abundances indicate uneven changes in the genetic representation of these steps. Denitrification genes occur in different combinations among microbial genomes, allowing different populations to carry distinct portions of the pathway (Graf et al., 2014). Selective changes among populations carrying these functions could therefore produce the N1 gene pattern. The ecological significance lies in the composition of the functional gene assemblage, beyond its combined abundance. D3-S also retained genetic potential for contrasting N outcomes. Both *nirK* and *nosZ* remained more abundant than in the initial D3 soil, while the DNRA-associated genes *nrfA* and *nrfH* also exceeded their initial abundances (Figures 4h,i). The former represent steps toward gaseous N products, whereas the latter are associated with respiratory nitrite reduction to NH_4_^+^. Their joint occurrence indicates potential for both gaseous N formation and ammonium regeneration within the saturated soil community. This distinction matters for subsequent N availability: regeneration of NH_4_^+^ can retain N for microbial assimilation or later oxidation, while denitrification can transfer N to gaseous products. Isotope-based measurements in rewetted soils have demonstrated that moisture and carbon availability can alter the relative importance of nitrate reduction pathways with different consequences for N retention and loss (Friedl et al., 2018). The gene assemblage therefore provides a biochemical basis for considering alternative N fates under the same moisture condition.

Glycine produced a broader endpoint functional profile. Compared with CK, N3 contained higher combined abundances of genes assigned to nitrification, denitrification and DNRA, together with larger relative abundances of *Aromatoleum*, *Ammoniphilus* and *Pseudomonas* (Supplementary Figures S3d; Figures 4j–l). The supply of both organic carbon and nitrogen offers a resource-based explanation for these changes: organic N turnover can supply inorganic N substrates, while carbon metabolism can support reductive pathways. Glycine-induced changes in amino acid metabolism and C- and N-cycling transcripts in soil microbiomes support this connection between substrate composition and microbial resource acquisition (Varliero et al., 2024). The coexistence of oxidative and reductive genetic potential fits this broader range of possible N transformations. By comparison, N2 retained a substantial NH_4_^+^-N pool but contained lower abundances of *amoA*, *amoB*, *amoC* and *hao* than CK at day 45. A larger ammonium supply therefore coexisted with a smaller endpoint gene pool associated with ammonia oxidation, emphasizing the influence of the wider environmental and incubation conditions on microbial functional composition.

Across the day-0 and day-45 samples from D1, D2 and D3, the 10 most abundant nitrogen-cycling gene annotations showed common positive associations with NO_3_^−^-N, TDN and DON (Supplementary Figure S4a). The similar patterns among annotations representing different functions suggest that the size of the dissolved N pools covaried with broad differences in encoded nitrogen-cycling potential. Larger gene pools therefore coexisted with substantial remaining nitrate, highlighting the relevance of initial soil resources and the chemical conditions represented by the sampled soils. The comparatively weak associations with DOC and Eh suggest that these two variables alone provided limited explanatory power across the combined dataset. Within D3, positive associations with NO_2_^−^-N extended across all 10 annotations (Supplementary Figure S4b). The parallel patterns for *nirA*, *nirK* and *nrfA* linked this intermediate to genetic potential for assimilatory nitrite reduction, denitrification and DNRA, respectively. Their shared association with NO_2_^−^-N is consistent with substrate conditions supporting several possible nitrite fates within the microbial community. Among the selected annotations, norB alone showed nominally significant negative correlations with DOC and Eh. Lower remaining DOC and lower Eh thus coincided with greater representation of nitric oxide reduction potential, suggesting a possible connection between substrate turnover, redox conditions and the populations carrying this function.

The differing rankings of gene abundance and nitrate decline also have a temporal dimension. D3-R contained more genes assigned to nitrate reduction than D3-S at day 45, although D3-S showed the greater nitrate concentration decrease. Most of the pronounced nitrate changes occurred between days 1 and 15, before the endpoint community experienced several additional weeks of changing carbon and nitrogen availability. Transcriptional regulation can alter N cycling activity without a proportional change in DNA-level gene abundance (Yoshida et al., 2012; Németh et al., 2014). Changes in expression, substrate availability and community composition could consequently contribute to the contrasting chemical and genetic patterns. Growth, survival and DNA persistence also influence the populations represented by DNA-based profiles (Wang et al., 2023b). The day-45 profiles therefore complement the chemical trajectories by showing how microbial functional potential differed after sustained exposure to contrasting saturation and substrate conditions.

### 5.4 Environmental implications and limitations

Higher soil saturation may create conditions that favor nitrate concentration decline during groundwater recovery, particularly where labile carbon is available. The contrast between nitrate persistence at 50% WFPS and larger decreases at 80 and 100% WFPS, together with the effect of glucose addition, supports this conditional environmental implication. Wetting associated with groundwater recovery could therefore reduce the soil nitrate pool available for transport when suitable carbon supplies and sufficient reaction time support a sustained concentration decline. Contact between nitrate-containing groundwater and organic soil under high water levels can support nitrate reduction, with restricted oxygen supply and organic carbon availability providing a mechanistic explanation (Roumelis et al., 2025). Nitrate attenuation documented in agricultural settings also supports evaluating this possible contribution to groundwater protection (Pan et al., 2024).

The extent of any benefit will depend on the carbon and nitrogen resources in the soils that become wetter. Contrasting nitrate trajectories among D1, D2 and D3 emphasize the relevance of initial soil composition, while the N0/N1 comparison demonstrates the influence of labile carbon supply within D3. Assessing soil conditions favorable for nitrate concentration decline therefore requires information on nitrate storage and carbon availability alongside water levels and soil moisture. Vertical heterogeneity can produce different nitrogen transformations as water levels change (Zhang et al., 2023). D3 provides an example of soil with comparatively high potential for nitrate concentration decline under the laboratory conditions of high saturation and suitable substrate supply. Its behavior highlights the possible importance of carbon-bearing materials in the lower vadose zone when evaluating the water quality consequences of groundwater recovery. Water movement and soil-water contact time will determine whether local concentration decreases translate into lower nitrate export. Deep vadose zones can store nitrate accumulated over long periods, creating a reservoir available for subsequent mobilization (Wang et al., 2023a). Infiltration and groundwater recharge influence how rapidly this stored N moves through the subsurface (Green et al., 2018; Turkeltaub et al., 2018). Groundwater recovery in the North China Plain makes the fate of these existing soil N stores increasingly relevant (Xu et al., 2026). Changes in water level can modify oxygen supply, microbial communities and N transformations while also redistributing solutes (Zuo et al., 2023). Groundwater nitrate concentrations will consequently reflect the timing of biogeochemical changes relative to transport, mixing and continuing nitrogen inputs.

Nitrogen form and timing add a further dimension to this assessment. Persistent NH_4_^+^-N after ammonium addition and successive inorganic N maxima following glycine addition show that changes in reduced and organic N pools can accompany temporary nitrate accumulation under high saturation. Biodegradable DON can act as an inorganic N precursor while its associated carbon modifies the conditions supporting nitrate reduction (Liu et al., 2022, 2023). Joint measurements of NO_3_^−^-N, NH_4_^+^-N, DON and DOC would help characterize these changes as groundwater levels recover. Focusing observations on periods of changing soil moisture would also help align chemical and microbial measurements with the onset of substrate and redox changes. Combined with measurements of microbial activity and N products, such observations could clarify when wetting favors declining nitrate availability and when other N pools sustain nitrate concentrations.

The experimental design limits extrapolation to these field processes. Static WFPS treatments represent a proxy for soil wetting and omit a moving groundwater boundary, hydraulic connectivity, leaching and water-level fluctuations; sieving, repacking and laboratory gas exchange also alter soil conditions. Sampling a single profile leaves depth and initial soil properties confounded, while substrate comparisons cover only D3 at 100% WFPS and include unequal carbon inputs from glucose and glycine. Without direct oxygen measurements, 100% WFPS does not establish uniform anoxia. The absence of gaseous N measurements, isotope tracing and a complete N balance leaves the contributions of denitrification, DNRA, microbial assimilation and other pathways unresolved. DOC decline likewise integrates several carbon exchanges rather than quantifying carbon use by a particular N transformation pathway. Initial and day-45 microbial profiles characterize community composition and encoded functional potential, with limited temporal correspondence to the earlier chemical changes. Kinetic parameters describe concentration trajectories, and comparisons with CK do not isolate the fate of added N. Field observations and experiments under realistic water flow are needed to establish when the observed potential for nitrate concentration decline contributes to sustained groundwater-quality improvement.

## 6 Conclusion

Increasing saturation enhanced nitrate concentration decline in soils collected from three depths of a single vadose-zone profile, accompanied by lower Eh and greater DOC decline. D3 soil, which had the highest initial DOC concentration, showed the largest proportional nitrate decline at 100% WFPS. Glucose addition further increased the extent and early rate of nitrate concentration decline in this soil, demonstrating the influence of labile carbon supply under saturated conditions. Nitrogen inputs produced contrasting temporal patterns: ammonium remained the dominant dissolved N form after ammonium addition, whereas glycine addition produced an early DON decline followed by successive ammonium, nitrite and nitrate peaks. Changes in bacterial community composition and day-45 functional gene profiles revealed differences in nitrogen-cycling potential among treatments. The contributions of individual transformation pathways remain unresolved without gaseous N measurements or isotope tracing. These findings distinguish the influence of carbon supply on nitrate persistence from the influence of substrate composition on the sequence and timing of dissolved N changes. Where labile carbon and sufficient reaction time support nitrate concentration decline, wetting associated with groundwater recovery may reduce soil nitrate available for transport and thereby help limit nitrate delivery to groundwater.

## Data Availability

The original contributions presented in the study are included in the article/Supplementary material, further inquiries can be directed to the corresponding author/s.
