# Supplementary material for: Microcosm evaluation of saturation and labile carbon controls on nitrate persistence in vadose zone soils
Source: Front Microbiol. 2026 Oct 28;17:1975842. doi: 10.3389/fmicb.2026.1975842 (PMC13751607; doi:10.3389/fmicb.2026.1975842)
Supplement: Supplementary file 1 [file Table_1.DOCX]

Supporting information

Saturation and substrate availability shape nitrate dynamics in vadose zone soils

Liangyao Chen^a^, Xiang Liu^a*^, Zhaohuan Li^a^, Han Li^a^, Yunqiang Xu^a^

^a^ School of Environment, Tsinghua University, Beijing 100084, China

Address Correspondence:

Xiang Liu

School of Environment, Tsinghua University

Haidian District, Beijing, 100084, P.R. China

Tel:+86-10-62783085

Email: x.liu@tsinghua.edu.cn

Figure S1. Changes in (a) Eh, pH and (b) dissolved organic carbon under different WFPS levels.

Figure S2. Changes in (a) Eh, pH and (b) dissolved organic carbon under different substrate treatments.

Figure S3. Bacterial community composition under different WFPS and substrate treatments. (a) WFPS treatments, phylum level. (b) WFPS treatments, genus level. (c) Substrate treatments, phylum level. (d) Substrate treatments, genus level.

Figure S4. Spearman correlations between the ten most abundant nitrogen-cycling gene annotations and environmental and dissolved nitrogen variables. (a) The saturation dataset, comprising the day 0 soils D1, D2 and D3 and their nine WFPS treatment groups on day 45. (b) The D3 substrate dataset, comprising the day 0 soil and CK, N0, N1, N2 and N3 on day 45.

Table S5. P values for exploratory Spearman correlations between the ten most abundant nitrogen-cycling gene annotations and environmental and dissolved nitrogen variables.

A. Saturation dataset corresponding to Figure S4a

| Dissolved nitrogen variables | | | | | |
| --- | --- | --- | --- | --- | --- |
| Gene | NO_3_^-^-N | NO_2_^-^-N | NH_4_^+^-N | TDN | DON |
| napA | 0.00126 | 0.08444 | 0.33384 | 0.00080 | 0.00582 |
| narG, narZ, nxrA | 0.00085 | 0.08444 | 0.26420 | 0.00052 | 0.00291 |
| narH, narY, nxrB | 0.00094 | 0.08444 | 0.28117 | 0.00058 | 0.00356 |
| nasC, nasA | 0.00182 | 0.10824 | 0.38259 | 0.00128 | 0.00541 |
| nasD, nasB | 0.00145 | 0.04984 | 0.48057 | 0.00147 | 0.03704 |
| nirA | 0.00081 | 0.08444 | 0.24788 | 0.00050 | 0.00291 |
| nirK | 0.00099 | 0.08444 | 0.28117 | 0.00062 | 0.00432 |
| norB | 0.00089 | 0.08444 | 0.26978 | 0.00055 | 0.00329 |
| nosZ | 0.00081 | 0.08444 | 0.25505 | 0.00050 | 0.00279 |
| nrfA | 0.00104 | 0.09780 | 0.25145 | 0.00058 | 0.00145 |
| Environmental variables | | | | | |
| Gene | DOC | Eh | pH | Moisture | C/N |
| napA | 0.51529 | 0.29113 | 0.85459 | 0.73541 | 0.00152 |
| narG, narZ, nxrA | 0.66270 | 0.33623 | 0.58364 | 0.68666 | 0.00085 |
| narH, narY, nxrB | 0.62153 | 0.31732 | 0.67716 | 0.69874 | 0.00104 |
| nasC, nasA | 0.55342 | 0.24991 | 0.83862 | 0.81665 | 0.00217 |
| nasD, nasB | 0.24309 | 0.26077 | 0.74428 | 0.71700 | 0.00152 |
| nirA | 0.65676 | 0.33410 | 0.60076 | 0.68064 | 0.00099 |
| nirK | 0.57575 | 0.29706 | 0.79426 | 0.70481 | 0.00145 |
| norB | 0.65676 | 0.34052 | 0.57234 | 0.69269 | 0.00081 |
| nosZ | 0.66270 | 0.33197 | 0.59789 | 0.68064 | 0.00089 |
| nrfA | 0.81033 | 0.32147 | 0.57234 | 0.70481 | 0.00138 |

B. D3 substrate dataset corresponding to Figure S4b

| Dissolved nitrogen variables | | | | | |
| --- | --- | --- | --- | --- | --- |
| Gene | NO_3_^-^-N | NO_2_^-^-N | NH_4_^+^-N | TDN | DON |
| napA | 0.82251 | 0.01590 | 0.16003 | 0.91085 | 0.73576 |
| narG, narZ, nxrA | 0.95537 | 0.00214 | 0.16003 | 1.00000 | 0.82251 |
| narH, narY, nxrB | 0.95537 | 0.00214 | 0.16003 | 1.00000 | 0.82251 |
| nasC, nasA | 0.91085 | 0.01086 | 0.13896 | 0.95537 | 0.69324 |
| nasD, nasB | 0.91085 | 0.01086 | 0.13896 | 0.95537 | 0.69324 |
| nirA | 0.53092 | 0.00003 | 0.10173 | 0.45564 | 0.95537 |
| nirK | 0.45564 | 0.00027 | 0.11953 | 0.49273 | 1.00000 |
| norB | 0.82251 | 0.03900 | 0.11953 | 0.69324 | 0.38512 |
| nosZ | 0.91085 | 0.00410 | 0.20703 | 0.91085 | 0.82251 |
| nrfA | 0.41975 | 0.04963 | 0.20703 | 0.49273 | 0.49273 |
| Environmental variables | | | | | |
| Gene | DOC | Eh | pH | Moisture | C/N |
| napA | 0.05799 | 0.08556 | 0.62654 | 0.00201 | 0.28940 |
| narG, narZ, nxrA | 0.16003 | 0.18272 | 0.86492 | 0.02082 | 0.38512 |
| narH, narY, nxrB | 0.16003 | 0.18272 | 0.86492 | 0.02082 | 0.38512 |
| nasC, nasA | 0.07099 | 0.10173 | 0.62654 | 0.00385 | 0.31989 |
| nasD, nasB | 0.07099 | 0.10173 | 0.62654 | 0.00385 | 0.31989 |
| nirA | 0.28940 | 0.26040 | 0.90977 | 0.07099 | 0.23294 |
| nirK | 0.26040 | 0.23294 | 0.90977 | 0.05799 | 0.20703 |
| norB | 0.04653 | 0.04653 | 0.52577 | 0.00385 | 0.31989 |
| nosZ | 0.10173 | 0.13896 | 1.00000 | 0.02082 | 0.28940 |
| nrfA | 0.31989 | 0.26040 | 0.68966 | 0.08556 | 0.73576 |

Notes: The ten gene annotations with the highest total abundances were selected separately for each dataset. Part A includes the day 0 soils D1, D2 and D3 and their nine WFPS treatment groups on day 45. Part B includes three individual D3 replicates on day 0 and five substrate treatments on day 45. Combined gene names identify shared KEGG annotations.
